# Elucidating the Interaction between Pyridoxine 5′-Phosphate Oxidase and Dopa Decarboxylase: Activation of B6-Dependent Enzyme

**DOI:** 10.3390/ijms24010642

**Published:** 2022-12-30

**Authors:** Mohammed H. AL Mughram, Mohini S. Ghatge, Glen E. Kellogg, Martin K. Safo

**Affiliations:** Department of Medicinal Chemistry and the Institute for Structural Biology, Drug Discovery, and Development, School of Pharmacy, Virginia Commonwealth University, Richmond, VA 23298, USA

**Keywords:** B6 enzymes, PLP transfer, protein–protein interactions, dopa decarboxylase, pyridoxine 5′-phosphate oxidase, protein–protein docking

## Abstract

Pyridoxal 5′-phosphate (PLP), the active form of vitamin B6, serves as a cofactor for scores of B6-dependent (PLP-dependent) enzymes involved in many cellular processes. One such B6 enzyme is dopa decarboxylase (DDC), which is required for the biosynthesis of key neurotransmitters, e.g., dopamine and serotonin. PLP-dependent enzymes are biosynthesized as apo-B6 enzymes and then converted to the catalytically active holo-B6 enzymes by Schiff base formation between the aldehyde of PLP and an active site lysine of the protein. In eukaryotes, PLP is made available to the B6 enzymes through the activity of the B6-salvage enzymes, pyridoxine 5′-phosphate oxidase (PNPO) and pyridoxal kinase (PLK). To minimize toxicity, the cell keeps the content of free PLP (unbound) very low through dephosphorylation and PLP feedback inhibition of PNPO and PLK. This has led to a proposed mechanism of complex formation between the B6-salvage enzymes and apo-B6 enzymes prior to the transfer of PLP, although such complexes are yet to be characterized at the atomic level, presumably due to their transient nature. A computational study, for the first time, was used to predict a likely PNPO and DDC complex, which suggested contact between the allosteric PLP tight-binding site on PNPO and the active site of DDC. Using isothermal calorimetry and/or surface plasmon resonance, we also show that PNPO binds both apoDDC and holoDDC with dissociation constants of 0.93 ± 0.07 μM and 2.59 ± 0.11 μM, respectively. Finally, in the presence of apoDDC, the tightly bound PLP on PNPO is transferred to apoDDC, resulting in the formation of about 35% holoDDC.

## 1. Introduction

Vitamin B6, arguably the most important vitamin, has six forms, including the primary forms pyridoxal (PL), pyridoxine (PN), and pyridoxamine (PM), and their phosphorylated derivatives PLP, PNP, and PMP, respectively [1,2]. PLP serves as a cofactor for approximately 180 B6-dependent (PLP-dependent) enzymes involved in several essential cellular processes, including racemization, decarboxylation, transamination, elimination, and others, which are responsible for neurotransmitter production, heme biosynthesis, nucleic acid production, sphingomyelin synthesis, and glucose metabolism, etc. [3,4]. Examples of PLP-dependent enzymes include serine hydroxymethyltransferase (SHMT), aspartate aminotransferase (AAT), glutamate decarboxylase (GAD), DOPA decarboxylase (DDC) or aromatic l-amino acid decarboxylase (AADC), threonine aldolase, and serine racemase. B6-dependent enzymes are biosynthesized as apo-B6 enzymes and then converted to the catalytically active holo-B6 enzymes, mostly by covalently binding to PLP as an aldimine to an active site lysine (Lys) residue [5,6]. Humans depend on a salvage pathway involving pyridoxal kinase (PLK), pyridoxine 5′-phosphate oxidase (PNPO), and pyridoxal phosphatase to synthesize PLP from the primary vitamers, PNP and PMP, and/or to recycle PLP during protein turnover [2,5,7].

Pathogenic mutations in the B6-salvage enzymes that lead to cellular deficiency of PLP or B6-dependent enzymes that impact on their activity are known or suspected to contribute to several pathologies, especially neurological ones [5,7,8,9,10]. One of the most well documented is PNPO-dependent neonatal epileptic encephalopathy (NEE), which is linked to reduced PLP in the cell as a result of several natural mutations in PNPO that lead to null or reduced enzymatic activity [7,9,10,11]. PLP is a very reactive compound and can potentially form aldimines with free amino groups on non-B6 proteins, disrupting their function [2]. To avoid such toxicity, the cell maintains a very low concentration of free PLP (unbound) through dephosphorylation to PL by pyridoxal phosphatase, as well as by PLP feedback inhibition of PNPO and PLK [5,12,13,14]. This creates an intriguing question of how a low concentration of cellular free PLP converts dozens of competing apo-B6 enzymes to their active holo-forms. Several studies, e.g., those on biophysical and biochemical binding and PLP transfer kinetics, suggest that the cell solves this problem by shielding the PLP through a complex formation between PNPO or PLK and apo-B6 enzymes for productive transfer of PLP from the former to the latter. For example, the Churchich group, using fluorescence spectroscopy (FP), affinity chromatography, and phosphatases as a trapping agent, showed that PLK forms a complex with AAT (dissociation constant of 3 µM), and that the trapping agent did not inhibit PLP transfer to the apo-B6 enzyme [15]. Another study by Cheung et al. using FP and surface plasmon resonance (SPR) analyses showed that PLK binds AAT and GAD with dissociation constants of 4.9 µM and 2.7 µM, respectively [16]. A more recent study by our group using FP also showed that human PNPO (hPNPO) forms interactions with several B6 enzymes, including rabbit cytosolic SHMT, *E. coli* SHMT, *E. coli* L-threonine aldolase, and *E. coli* AAT with dissociation constants ranging from 0.3 to 12.3 μM, with concomitant transfer of a tightly bound PLP from PNPO to activate apo-SHMT to the holo-form [8]. These studies, however, although suggestive of complex formation prior to the transfer of PLP from the donor enzymes to the acceptor enzymes, do not address on an atomic level how the salvage and PLP-dependent enzymes recognize and interact with each other.

DDC (or AADC) catalyzes the conversion of aromatic amino acids to their corresponding amines during the synthesis of a variety of essential neurotransmitters, e.g., decarboxylation of L-DOPA into dopamine (DA), 5-HTP to serotonin, L-tyrosine to tyramine, and tryptophan to tryptamine [17,18]. As a result of its essential involvement in neurotransmitter biosynthesis, DDC has become a major target for research on Parkinson’s disease, depression, and other neurological illnesses [18,19,20,21,22,23,24]. Mutations in the DDC gene can lead to a rare neurotransmitter metabolic disorder known as aromatic L-amino acid decarboxylase deficiency (AADCD) (OMIM: 608643) [25,26,27]. It is a well-known genetic disorder in DDC that leads to serotonin and catecholamine deficiency, resulting in significant developmental impairment, as well as lifelong motor, behavioral, and autonomic symptoms, such as oculogyric crises (OGC), sleep disorders, and mood disorders [25,28]. More than 20 mutations in the DDC gene have been characterized and identified as causing AADCD [29,30]. Some of these mutations, e.g., S147R, G102S, F309L, and A275T, are located in the active site and are likely to impair the integrity of the active site, resulting in decreased PLP binding. Other mutations, e.g., Y79C, H70T, H72Y, and T69M, located on a loop region, are suggested to impair proper conformational change during PLP-induced apo–holo transition. Mutations such as L38P and A110Q are in the dimer interface and likely to perturb the dimerization configuration process [30,31,32]. Some other mutations, which are neither located in the dimerization nor the catalytic site or loop region, could potentially affect PLP transfer through physical binding with the salvage enzymes. Clinical phenotypes of patients with AADCD are well established; nevertheless, enzymatic phenotypes of AADCD patients are mostly unknown and molecular mechanisms by which some of these mutations result in DDC deficiency remain unclear and ambiguous.

PNPO, a flavin-mononucleotide (FMN)-dependent enzyme, catalyzes PLP formation by oxidizing the 4′-hydroxyl group of PNP or the 4′-amino group of PMP [5,6]. In addition to the catalytic active site where PLP is synthesized, PNPO is known to have a strong affinity for the cofactor at a non-catalytic secondary binding site (allosteric site) [8,14,33]. A crystallographic study on the *E. coli* PNPO enzyme (ePNPO) identified a solvent-protected allosteric site with a tightly bound PLP [34]. An earlier crystallographic study with the unliganded human enzyme (hPNPO) identified the same allosteric site with a bound phosphate, with the two molecules overlapping. The tightly bound PLP has been suggested to play several physiological roles, including allosteric feedback inhibition of PNPO [14], as well as a source for activating the apo-B6 enzyme, as it easily transfers to apo-PLP-dependent enzymes [8]. It is interesting to note that although the tightly bound PLP does not easily dissociate from the enzyme, even during size exclusion chromatography or dialysis, the presence of the apo-B6 enzyme induces the transfer of a fraction of the bound PLP to activate the apo-B6 to the holo-enzyme [8]. As noted above, it has been suggested that the transfer of the PLP is preceded by interaction between PNPO and apo-B6 enzymes, although no such complex has been characterized on the atomic level. In this study, we used molecular modeling, biophysical binding, and PLP transfer studies to predict the complex formation between PNPO and DDC, as well as to gain insight into how apo-B6 enzymes are activated to their catalytically active holo-forms.

## 2. Results and Discussion

### 2.1. Predicting Interactions between hPNPO and DDC Using Molecular Modeling

A proposed mechanism of B6 enzyme activation involves complex formations between the salvage and apo-B6 enzymes for a productive transfer of PLP from the former to the latter [2,5,8,12]. A long-standing question that is yet to be answered is how the salvage enzymes recognize and interact with the B6 enzymes. In the absence of an experimentally determined complex structure, we used molecular docking/dynamics simulation studies to determine potential complex formation between hPNPO (PDB 1NRG) and DDC. DDC was chosen for the study since unlike most B6 enzymes, for which only the holo-form structures are known, the mammalian DDC crystal structure is available in both the apo-form from human (PDB 3RCH, 3RBF, and 3RBL) and the holo-form from pig kidney (PDB 1JS3). The 2 sources of enzymes have 90% sequence homology [20,35]. Interestingly, the crystal structures of DDC in the apo-form (open conformation) and the holo-form (closed conformation) show significant conformational differences, implying that molecular modeling studies on the interaction between the B6-salvage and B6-dependent enzymes should encompass both conformations. All 3 reported apoDDC structures were initially docked to hPNPO using the Cluspro 2.0 server [36,37], and since there were no discernible differences between the putative complexes, 3RCH, which had the best resolution (2.85 Å), was subsequently used for further docking studies. It is notable that several amino acid residues are absent in both the reported apoDDC (Gly102-Ala107, Thr323-Arg355) and holoDDC (Leu328-Gly339) structures, likely due to disorder in these regions [35]. These missing residues were modeled and refined to obtain apoDDC and holoDDC structures with all amino acid residues as described in Section 3.

Interestingly, the docking studies between hPNPO and apoDDC or holoDDC (crystallographic structures with missing residues) showed no direct interactions between the active sites of the two interacting proteins. Rather, the formed interface involved the allosteric PLP binding site of PNPO and the active site of DDC, supporting a long-held view that the activation of apo-B6 enzymes in part involves the transfer of the tightly bound PLP (PNPO●PLP) to apo-B6 enzymes [8,33,34]. Crystallographic studies with the *E. coli* enzyme identified the tightly bound PLP at an allosteric site, which is solvent-protected [34]. Earlier crystallographic studies with the unliganded human enzyme also identified a bound phosphate at the allosteric PLP site (PDB 6H00). Even though the PLP and phosphate binding sites of *E. coli* and hPNPO, respectively, overlap, it is notable that the two binding sites are not conserved. Additionally, of interest is that, while the docking study showed PNPO to recognize just one site of the asymmetric dimeric structure of apoDDC, the complex with the holoDDC (symmetric dimer) revealed a favorable binding for both PLP sites. This observation in part may explain the partial activation of apo-B6 enzymes by either PNPO•PLP or PLK•PLP, as previously reported [8,12], and apoDDC in this work (*vide infra*). It is also conceivable that the partial activation may be partly due to the establishment of the equilibrium of PLP binding to the donor and acceptor proteins, as not all PLP is expected to be transferred from the donor protein to the acceptor protein.

**Figure 1:**
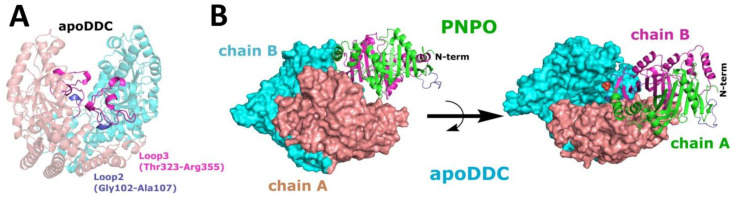
PNPO•apoDDC complex structure predicted using Cluspro 2.0. (**A**) ApoDDC structure model with an entire amino acid chain (3RCH-AF). (**B**) Top predicted structure of the PNPO•apoDDC complex.

**Figure 2:**
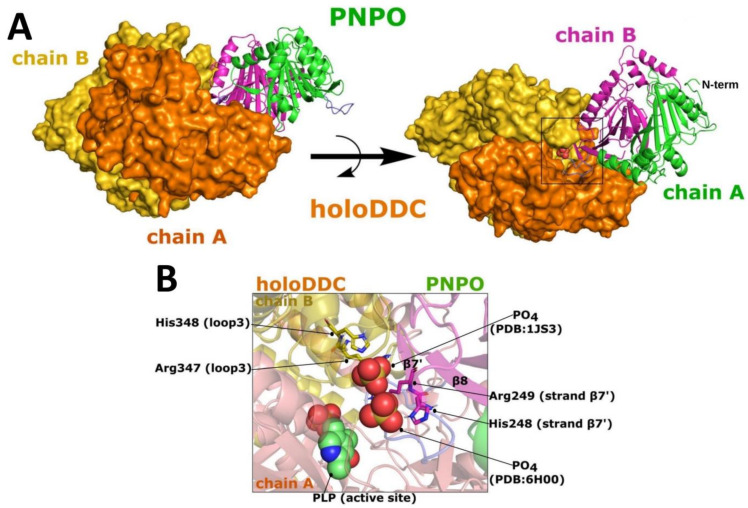
PNPO•holoDDC complex structure predicted using Cluspro 2.0. (**A**) Top predicted structure of the PNPO•holoDDC complex. (**B**) Superimposition of the crystal structures PNPO (PDB 6H00) and holoDDC (PDB 1JS3) over the PNPO•holoDDC complex model.

**Figure 3:**
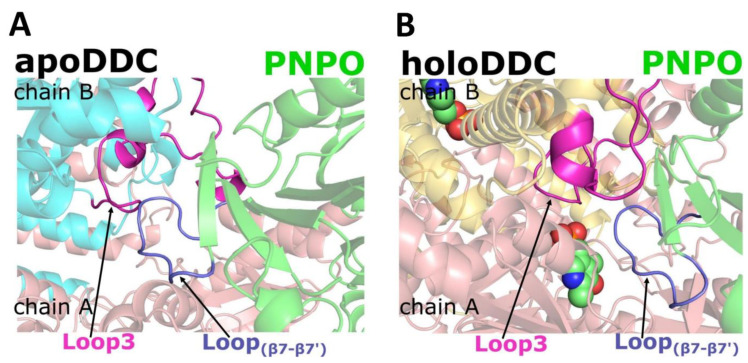
Hot loops predicted at the putative interface of (**A**) PNPO•apoDDC and (**B**) PNPO•holoDDC complexes.

As pointed out above, both apoDDC (PDB 3RCH) and holoDDC (PDB 1JS3) structures are missing several residues that include Gly102-Ala107 and Thr323-Arg355 from the apoDDC, and Leu328-Gly339 from the holoDDC. The residues Thr323-Arg355 are part of a loop3 structure. We decided to model these missing residues and use the models for docking studies similar to those described above (see Materials and Methods for details). A computationally predicted apoDDC AlphaFold structure from the AlphaFold protein structure database [38] was found to be the most reliable model for this missing portion of the apo-protein, which was extracted and inserted into the crystal structure of 3RCH to create a model with an entire amino acid residue present, referred to as 3RCH-AF (Figure 1A). Docking PNPO to the 3RCH-AF model revealed a significant improvement in the formed interface and shape complementarity, and consequently, an improvement in the HINT scores of complex interface residues, i.e., from 2032 (~3.95 kcal mol^−1^) to 8650 (~16.8 kcal mol^−1^) for the most populous cluster (Figure 1B). HINT (hydropathic interaction) is an empirical forcefield based on experimental logP_o/w_ values for interaction classification and scoring [39,40]. Notably, the 2 most populous clusters (both with a similar binding mode) account for 9.7% of the total generated solutions after modeling missing residues, compared to 14.7% when docked to the crystal structure with the missing residues incorporated (see Supporting Information Tables S1 and S2). Importantly, all top (most populated) clusters for the PNPO•apoDDC complex demonstrated that the surface loop (residues Gly235-Thr247) that connects strands β7 to β7′ on PNPO (termed loop_β7-β7′_) was anchored to the dimeric interface of DDC, implying the possibility of transient “loop-mediated” interactions between the two proteins (Figure 1B). This protein–protein association involving the allosteric PLP binding site of PNPO and the active site of apoDDC was also observed for the PNPO•holoDDC complex (Figure 2), as discussed further below. Interestingly, the crystal structure of the human PNPO (PDB 1NRG) shows the β7′ strand, along with a portion of a turn and loop_β7-β7′_, to be unique in the human enzyme as these structures are absent in the *E. coli* and yeast structures. Notably, loop_β7-β7′_ of PNPO was observed to be in direct contact with the loop3 region (residues 323–357) of both apoDDC and holoDDC structures (Figure 3A,B).

Likewise, modeling and refinement of the missing residues (Leu328-Gly339) in the holoDDC (PDB 1JS3) was carried out as described in the Methods and Materials. Unlike the apoDDC that was missing loop3 (Thr323-Arg355), loop3 in holoDDC was relatively well structured and buried, although it exhibited some degree of disorder, as shown by the missing electron density of residues 328–339. The PNPO•holoDDC clusters 0 and 1 account for 15.2% of the total generated solutions after modeling missing residues, compared to only 11.3% when docked to the crystal structure with the missing residues. Similarly, the HINT scores for clusters 0 and 1 improved from 2222 (~4.31 kcal mol^−1^) and 2401 (~4.66 kcal mol^−1^) to 4746 (~9.21 kcal mol^−1^) and 4756 (~9.23 kcal mol^−1^), respectively, without affecting the generated interface (Supporting Information Tables S3 and S4).

It is interesting to note that the holoDDC structure (PDB 1JS3) has a bound phosphate molecule at the putative contact interface with PNPO (Figure 2B). In the hPNPO structure (PDB 6H00), a phosphate is bound to the PLP allosteric site, which also forms part of the putative contact interface (Figure 2B). Note that the ePNPO crystal structure (PDB 6YMH) actually shows a bound PLP at the allosteric site, which overlaps the bound phosphate in the hPNPO structure (PDB 6H00) [34]. The residues surrounding these surface phosphate binding sites are His248 and Arg249 in the strand β7′ region of the PNPO, and Arg347 and His348 in the loop3 region of holoDDC (Figure 2B). Notably, in the docked complexes, these two phosphates from PNPO and DDC lie adjacent to each other. Thus, it is likely that the phosphate binding sites serve as a marker for the first ligand recognition site of PLP at the interfacial domain. With this in mind, DDC gene missense mutations c.1039C > G (p.Arg347Gly), c.1039C > T (p.Arg347Trp), and c.1040G > A (p.Arg347Gln) have been linked to AADCD (OMIM #608643) [26,30,41]. We speculate that Arg347X mutations may lead to weakening of the complex interaction and/or misalignment of the two complexes, preventing productive transfer of PLP. This residue is a candidate for site-directed mutagenesis study. Voltattorni’s group, on the other hand, investigated loop3 variants and concluded that this loop is also essential for proper substrate binding and orientation [30]. Based on cluster size, conservation of the created complex, and HINT scores, the best models for PNPO•holoDDC and PNPO•apoDDC complexes were selected (Figure 1B and Figure 2A) for further refinement using molecular dynamics (MD) simulations [42,43].

**Figure 4:**
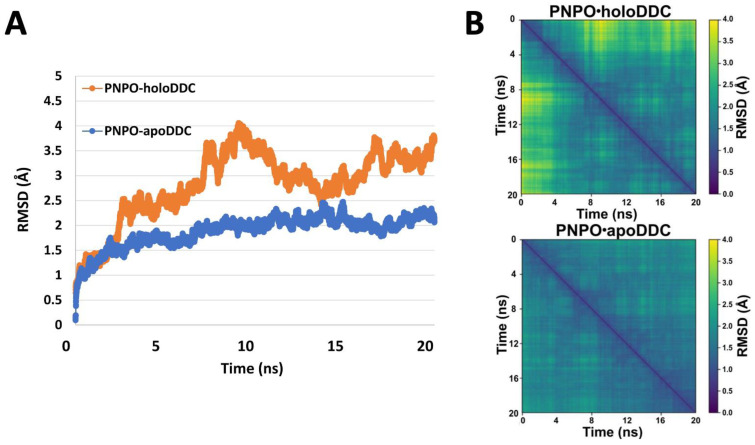
MD simulation of PNPO•holoDDC and PNPO•apoDDC putative complexes. (**A**) RMSD analysis (Å) for the PNPO•holoDDC (blue) and PNPO•apoDDC (orange) complexes during MD simulation. (**B**) Heat maps representing pairwise RMSD (Å) calculated for the PNPO•holoDDC (above) and PNPO•apoDDC (below) complexes.

**Figure 5:**
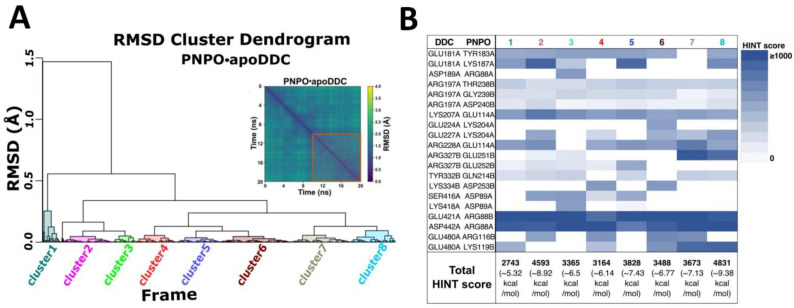
Hierarchical clustering and scoring of MD trajectories for the PNPO•apoDDC putative complex. (**A**) Hierarchical clustering analysis for the last 10 ns of trajectories (500 frames). (**B**) Frequencies and HINT scores of interacting key amino acid residues at the protein–protein interface with respect to clusters (~515 HINT unit = −1 kcal mol^−1^).

**Figure 6:**
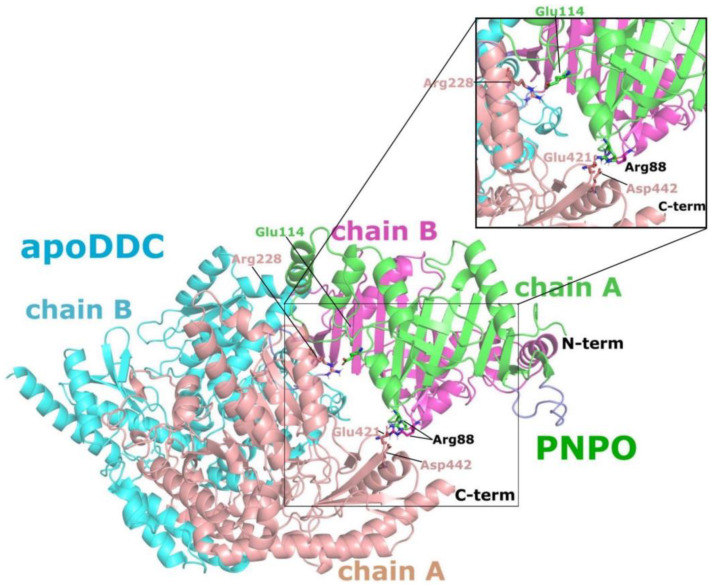
Key residues predicted by HINT scores and in silico alanine scanning mutagenesis for the PNPO•apoDDC complex.

An all-atom MD simulation with NAMD 2.9 [43,44] was used to refine the selected PNPO•apoDDC and PNPO•holoDDC complexes. To measure the quality and convergence of the MD trajectories, backbone root mean square deviation (RMSD) and pairwise RMSD values relative to the initial structures were determined, as shown in Figure 4. Both complexes were shown to be well maintained over the duration of the 20 ns simulation; i.e., following an increase in RMSD within the first 2 ns, MD trajectories stabilized with average RMSD values of 1.96 ± 0.21 Å and 2.99 ± 0.48 Å for PNPO•apoDDC and PNPO•holoDDC, respectively (Figure 4). The MD trajectories of the last 10 ns (500 frames) of the PNPO•apoDDC complex were clustered into unique structural conformations (Figure 5A) using the RMSD dendrogram with clustering annotation (Bio3D package in R [45]). This allowed the energetic contributions of interfacial residues to be evaluated using the HINT score function (Figure 5B), as well as in silico alanine mutagenesis studies to estimate the impact of interfacial residue alanine mutations on the binding free energy (ΔG) of the complex (see Supporting Information Table S5). In silico alanine-scanning mutagenesis is an effective and common approach to identifying hotspot residues; important contributions are made by key residues whose replacement with alanine results in a binding energy loss of ΔΔG ≥ 1 kcal mol^−1^ [46,47]. Several putative electrostatic interactions appear to be important in holding these two enzymes together. Most prominent are the residues E114 and R88 from PNPO that appear to form strong interactions with the apoDDC residues K207/R228 and E421/D442, respectively (Figure 5B and Figure 6). In silico alanine scanning (Robetta server [47]) showed that PNPO–R88 and PNPO–E114 would have the largest impact on the stability of the complex (see Supporting Information Table S7); i.e., the average ΔΔG_calc_ values were 2.35 ± 1.23 kcal mol^−1^ and 1.1 ± 0.79 kcal mol^−1^ for PNPO–R88 and PNPO–E114, respectively. These residues were identified as possible candidates for site-directed mutagenesis experimental testing. Figure 6 displays the structure of the PNPO•apoDDC complex model, including the putative salt bridges formed between PNPO–R88 and apoDDC–E421/apoDDC–D442, and PNPO–E114 and apoDDC–R228 (initial structure). The PNPO•apoDDC complex model would be the target of the site mutation experiments, as we are interested in targeting the interactions that bring the two proteins together for subsequent PLP transfer. Interaction profiles of hydrogen bonding and salt bridges across protein–protein interfaces for both PNPO•apoDDC and PNPO•holoDDC complexes are accessible in the Supporting Information Tables S6 and S7.

### 2.2. PNPO Forms a Physical Complex with Both the apoDDC and holoDDC

Our docking/molecular dynamics simulation studies suggest potential interactions between PNPO and DDC that involve the allosteric PLP binding site on PNPO and the PLP binding site of DDC. We subsequently utilized two biophysical binding techniques, isothermal calorimetry (ITC) and surface plasmon resonance (SPR), to experimentally quantify the interactions between hPNPO protein and both the apo- and holoDDC proteins.

**Figure 7:**
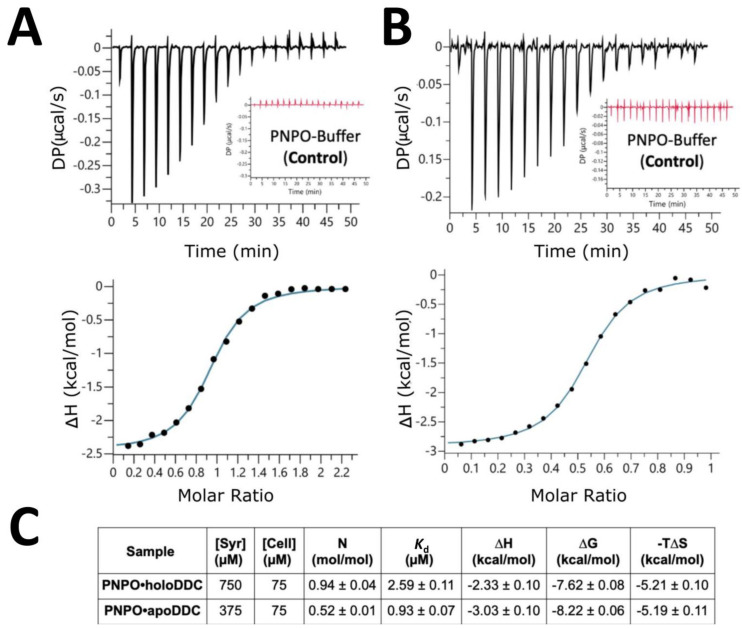
ITC thermodynamic analysis of PNPO•holoDDC and PNPO•apoDDC binding. Injection heats for titration of PNPO into (**A**) holoDDC and (**B**) apoDDC. (**C**) Thermodynamic parameters obtained from ITC measurements of PNPO•holoDDC and PNPO•apoDDC bindings. All titrations were run in at least triplicate.

The ITC binding titration for the purified recombinant hPNPO and DDC (both apo- and holoDDC) enzymes were carried out as described in Section 3. Both PNPO•holoDDC and PNPO•apoDDC complexes were satisfactorily characterized by ITC in terms of the binding interactions (Figure 7A,B, respectively). The thermodynamical parameters ΔG, ΔH, and ΔS for interactions between PNPO and holoDDC of −7.62 ± 0.08, −2.33 ± 0.10, and −5.21 ± 0.05 kcal mol^−1^, respectively, were obtained. Meanwhile, ΔG, ΔH, and ΔS values of −8.22 ± 0.06, −3.03 ± 0.10, and −5.19 ± 0.11 kcal mol^−1^, respectively, were obtained for the titration of PNPO and apoDDC (Figure 7C). Both titration studies provided an exothermic binding isotherm, with the enthalpy change being more negative for apoDDC than for holoDDC, likely due to the larger protein–protein interface in the case of apoDDC. Furthermore, PNPO displayed a roughly 3-fold-greater affinity toward apoDDC than holoDDC, i.e., *K*_d_ 0.92 ± 0.07 μM versus 2.59 ± 0.11 μM. The PNPO•holoDDC complex showed a stoichiometry close to 1 (0.94 ± 0.04), indicating that the dimeric PNPO binds to both active sites of the dimeric holoDDC. In contrast, the PNPO•apoDDC complex revealed a stoichiometry of 0.5 (0.52 ± 0.01), indicating that PNPO binds to just 1 site of the dimeric apoDDC. These observations are consistent with the modeling/docking studies described above that showed only one molecule of hPNPO is capable of binding to the asymmetric apoDDC dimer structure, while two molecules of PNPO demonstrated an equal recognition of both sites of the symmetric dimer of the holoDDC. Importantly, we were able to show that ITC can reliably characterize the binding of hPNPO and DDC in both apo- and holo-forms, which was also confirmed with the SPR technique.

The SPR experiment consisted of DDC immobilization on a biosensor surface (ligand sample), and PNPO in solution (analyte sample) [48]. Both the human DDC and PNPO proteins were recombinantly expressed and purified to homogeneity, as described in the Materials and Methods. The PLP-dependent enzyme holoSHMT was utilized as a positive control, as we have previously demonstrated its binding to B6-salvage enzymes [8]. In this experiment, the binding constant, *K*_d_, for PPI between hPNPO and holoDDC and holoSHMT, was determined to be 3.7 μM and 15.4 μM, respectively, using 7 different concentrations of PNPO, ranging from 100 μM to 1.56 μM, as shown in Figure S1. However, SPR was incapable of determining the binding affinity of hPNPO to apoDDC. While the signals from the control flow cell (without protein) were normal, the signals from the flow cell in which apoDDC was immobilized declined rapidly. When apoDDC interacted with PNPO, it is likely that the apoDDC underwent significant structural rearrangement (open–closed transition) due to the transfer of PLP from hPNPO to apoDDC on PPI. The negative control analyte, albumin, showed no effect, i.e., no binding, on the generated response unit.

### 2.3. PLP Transfer from PNPO●PLP Complex to apoDDC

**Figure 8:**
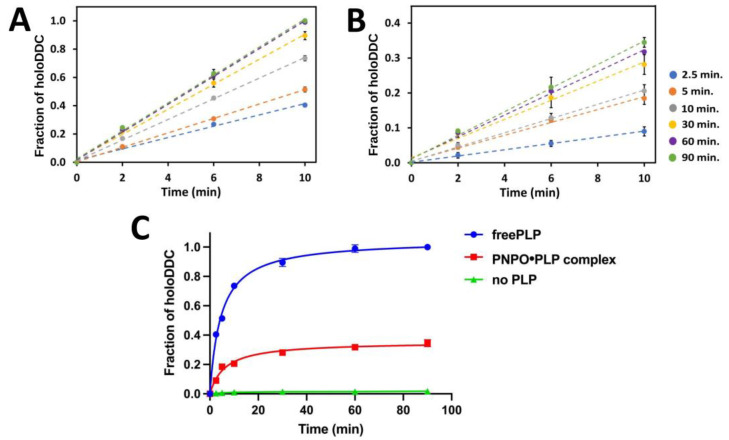
PLP transfer from PNPO•PLP to apoDDC. (**A**,**B**) Activation of apoDDC after 2.5, 5, 10, 30, 60, and 90 min of incubation with free PLP (**A**) and PNPO•PLP complex (**B**). (**C**) Final plot displaying dopamine formation at different activation time points. Data were fitted and analyzed using prism GraphPad software. The PLP transfer rate with free PLP, PNPO•PLP, or no PLP source is depicted by the blue, red, and green lines of fitted data, respectively.

The PLP produced under steady state by PNPO and PLK, as well as PLP tightly bound to PNPO (PNPO●PLP) or PLK (PLK●PLP) have previously been shown to activate apo-B6 enzymes to the holo-forms, and there is some evidence to suggest that the transfer of the PLP requires a complex formation between donor and acceptor enzymes, which would provide a safe and efficient pathway for productive activation of apo-B6 enzymes [8,12,15,16]. In this study, we used computational studies to model the putative complex between hPNPO and DDC, which interestingly suggested that the binding interface between the proteins involves the allosteric PLP tight binding site of PNPO and the active site of DDC, consistent with a long-held view that the tightly bound PLP plays role in PLP-dependent enzyme activation [8,12,33]. Following the computational and biophysical studies, we developed a new PLP transfer assay to study the capability of the tightly bound PLP transfer from hPNPO to activate apoDDC to the holoDDC form. The transfer assay is a spectrophotometric that monitors formation/activation of holoDDC indirectly by measuring product (dopamine) formation, as detailed in Section 3. One of the primary physiological reactions catalyzed by holoDDC involves decarboxylation of L-3,4-dihydroxyphenylalanine to form dopamine (L-DOPA). Lys303 at the active site of holoDDC forms an internal aldimine with the aldehyde group of PLP. The substrate L-DOPA replaces the Lys to form external aldimine with PLP, which is subsequently decarboxylated to form dopamine. Briefly, this assay involves two steps. First, the PLP binding step, in which apoDDC (50 µM) is mixed with an equimolar amount of free PLP (50 µM) or PNPO•PLP complex (50 µM) and incubated at 25 °C (dry bath incubator) for 0, 2.5, 5, 10, 30, 60, and 90 min. This step allows the formation of the enzymatically active holoDDC. Second, aliquots of these mixtures are diluted (100-fold) in a solution containing 2 mM L-Dopa in 100 mM potassium phosphate buffer, at pH 7.4 (substrate mixture). The L-Dopa is converted to dopamine by holoDDC. Dopamine formation at different time intervals (0, 2, 6, and 10 min intervals) using both the free PLP and PNPO•PLP complex was fitted into a linear equation. As shown in Figure 8A,B, R^2^ values ranging from 0.993 to 0.9997 for free PLP and 0.9878 to 0.9979 for the PNPO•PLP complex were obtained. The final PLP transfer plot revealed that in the presence of the PNPO•PLP complex, holoDDC reached ~35% of its activity when compared to an equal amount of free PLP, as seen in Figure 8C. Similar observations have been reported previously for apoSHMT, where ~25–40% of the tightly bound PLP was transferred when equimolar concentrations of PNPO•PLP or PLK•PLP were used [8,12].

## 3. Materials and Methods

### 3.1. Computational Studies for Predicting the Complex Structure between hPNPO and DDC

#### 3.1.1. Preparation of the Crystal Structures of hPNPO, holoDDC, and apoDDC

All crystal structures for the computational study were retrieved from the Protein Data Bank (PDB) [49]. Gaps due to missing electron density in both holoDDC (Leu328-Gly339) and apoDDC (Gly102-Ala107, Thr323-Arg355) were modeled using the MODELLER (MODELLER-II-Chimera GUI interface [50]) and AlphaFold protein database [38], respectively, with final structures prepared in SYBYL-x 2.1.1 (Tripos International, St. Louis, MO, USA). For holoDDC, a total of 100 models were generated, and the best models were inspected using DOPE (discrete optimized protein energy) and GA341 scores. The top 10 models were selected for further refinement using the GalaxyLoop server [51], where modeled regions were specified for refinement. MolProbity was utilized to assess the quality of models [52]. For apoDDC, AlphaFold prediction was found to generate the most reliable model for missing regions. Therefore, missing residues were extracted and inserted into the apoDDC crystal structure (3RCH), hydrogens were added, and the final structure (3RCH-AF) was minimized in SYBYL-x 2.1.1 with the Tripos forcefield.

#### 3.1.2. Molecular Docking

Molecular docking studies were performed with ClusPro 2.0 [36]. The enzymes apo- and holoDDC (receptor protein) were set at the coordinate system’s origin, and the various rotational and translational positions of the ligand (PNPO) were assessed at the specified level of discretization. The interaction energy between two proteins was represented by PIPER using the expression E = _w1_E_rep_ + _w2_E_attr_ + _w3_E_elec_ + _w4_E_DARS_, in which E_rep_ and E_attr_ are the repulsive and attractive contributions to the van der Waals interaction energy, respectively, while E_elec_ is an electrostatic energy factor. E_DARS_ is a pairwise structure-based potential that is built using “decoys as the reference state” (DARS). From the previous step, the 1000 rotation/translation combinations with the lowest scores are considered, which would be clustered with a C-alpha RMSD radius of 9 Å, resulting in 30 clusters for the 1000 poses with the lowest energy. From the preceding stage, 1000 rotation/translation combinations with the lowest scores are considered, which are clustered using a C-alpha RMSD radius of 9 Å, resulting in 30 clusters for the 1000 poses with the lowest energy. Using SYBYL-x 2.1.1 (Tripos International, USA), hydrogen atoms were added to the top 10 cluster models and subjected to Powell minimization (Tripos forcefield, with Gasteiger–Hückel charges and distance-dependent dielectric) to a gradient of 0.02 kcal mol^−1^ Å^−1^. Then, the HINT (hydropathic interaction) forcefield was applied to predict the free energy of the top 10 models.

#### 3.1.3. Molecular Dynamics Simulations

The chosen complexes of PNPO•apoDDC and PNPO•holoDDC were refined using all-atom molecular dynamics (MD) simulations. MD simulations were performed using the NAMD 2.9 package developed by the Theoretical and Computational Biophysics Group at the University of Illinois in Urbana-Beckman Champaign’s Institute for Advanced Science and Technology [43]. The forcefield used was CHARMM (Charmm-36) [53]. VMD 1.9.3 was used to prepare the system for MD simulations [42]. Using the psfgen module, coordinate (.pdb) and connectivity (.psf) files were generated for the final complexes followed by solvation in a cubic box of TIP3P water molecules with a minimum spacing of 9 Å between the box boundaries and the nearest solute atoms. The solvated system was then ionized with 0.15 M NaCl using the VMD autoionize plugin. All MD simulations were conducted in five sequential steps: (i) water minimization, (ii) entire system minimization, (iii) heating stage, (iv) NPT pre-production simulation, and (v) production simulation. First, 2000 steps of conjugate gradient minimization were applied to solvent molecules to ensure that water molecules were distributed evenly throughout the complex’s surface, followed by 20,000 steps of energy minimization of the entire system. Subsequently, gradual heating to 310 K was performed over a duration of 100 ps. In the first 2 rounds of equilibration, harmonic restraints were applied using restraint force constants of (5 kcal/(mol-Å)) under isothermal isobaric (NPT) ensemble for 1 ns, placed on proteins and the cofactor, in the case of holoDDC, and all other parameters remaining unchanged. In the following stage, the harmonic constraint was released, and the whole system was equilibrated for a further 1 ns using the NPT ensemble. The final production run was performed under the NPT ensemble, which simulated the whole system for 20 ns. All simulations were run on our GPU-based compute server, and molecular dynamic trajectories were analyzed using VMD [42], HINT score [40], Bio3D [45], and MDAnalysis [54] packages. All figures were generated using PyMOL (Version 2.0 Schrödinger, LLC., New York, NY, USA).

### 3.2. Subcloning, Expression, and Purification of DDC

DDC was produced by subcloning the coding sequence of the human wild-type dopa decarboxylase (DDC-OHu25359, GenScript, Piscataway, NJ, USA). ORF was cloned into the pET21d(+) plasmid using the NcoI and HindIII restriction sites. The plasmid construct was confirmed by DNA sequence analysis. The recombinant plasmid was then transformed into *E. coli* BL21(DE3)pLysS competent cells. The recombinant clones were grown in LB broth with ampicillin (100 μg/mL) at 37 °C until the OD600 was around 0.6–0.7, and then induced with 100 µM isopropyl-β-D-thiogalactopyranoside (IPTG). The cells were further grown at 30 °C for a further 14 h before being harvested and resuspended in a lysis buffer containing 50mM sodium phosphate, 300 mM NaCl, 10 mM imidazole, 50 µM PLP, and 0.2 mM PMSF, pH 8. The clear lysate was diluted and loaded on a 5 mL Histrap column equilibrated with 10-column volume of buffer A (50 mM potassium phosphate buffer pH 8, 300 mM NaCl, and 10 mM imidazole). Following this, a linear gradient of buffer B (50 mM potassium phosphate buffer pH 8, 300 mM NaCl, and 1 M imidazole) was applied (0–100% in 500 mL). DDC eluted as a single peak from the Ni2+ resin with imidazole at concentrations ranging from 100 to 300 mM. The purified DDC was dialyzed against 150 mM NaCl and 100 µM PLP in 50 mM potassium phosphate, pH 7.4, overnight. A second dialysis in the same buffer, without PLP, was performed for an additional 5 h. Purified DDC was concentrated using Amicon Ultra 10 concentrators (Millipore, Burlington, MA, USA), and the concentration determined using a εM of 1.3  ×  10^5^ M^−1^cm^−1^ at 280 nm.

### 3.3. Preparation of apoDDC

The enzyme holoDDC was incubated at room temperature for 3 h with 5 mM hydroxylamine in 0.5 M potassium phosphate buffer, pH 6.9, followed by overnight dialyzing against 50 mM potassium phosphate buffer, pH 7.4, containing 150 mM NaCl and 5% glycerol. A second dialysis was carried out for an additional 5 h in the same buffer without glycerol. The total conversion of holoDDC to apoDDC was validated using the DDC enzymatic activity assay in the presence and absence of the cofactor (PLP).

### 3.4. Expression and Purification of Human PNPO

The recombinant hPNPO expression, and purification was carried out as previously described by our group [6]. An overnight culture was used to inoculate 6 L of LB medium containing (40 μg/mL) of kanamycin. *E. coli* cells were grown at 37 °C with rotary shaking until OD600 reached 0.6–0.7, then induced with 100 µM isopropyl-β-D-thiogalactopyranoside (IPTG). The cells were further incubated at 18 °C for a further 14 h before being harvested and resuspended in a lysis buffer containing 50 mM sodium phosphate, 300 mM NaCl, 10 mM imidazole, 10 µM FMN, and 0.2 mM PMSF, pH 8. The clear lysate was diluted and loaded on a 5 mL Histrap column equilibrated with 10-column volume of buffer A (50 mM potassium phosphate buffer pH 8, 300 mM NaCl, and 10 mM imidazole) after centrifugation. Following this, 500 mL of a linear gradient of buffers A and B (50 mM potassium phosphate buffer pH 8, 300 mM NaCl, and 500 mM imidazole) was applied (0–100%). PNPO eluted as a single peak (yellow fractions) from the Ni^2+^ resin with imidazole at concentrations ranging from 100 to 300 mM. The purified PNPO was dialyzed overnight against 50 mM potassium phosphate, pH 7.4, containing 150 mM NaCl and 10 µM FMN. A second dialysis in the same buffer, without FMN, was performed for an additional 5 h. Purified PNPO was concentrated using Amicon Ultra 10 concentrators (Millipore), and the concentration was determined using the molar extinction coefficient of 76,760 M^−1^cm^−1^ at 280 nm.

### 3.5. Activity Assay of DDC

The enzymatic activity of DDC was determined as previously described by Sherald et al. [55], with modifications by Charteris and John [56]. The enzyme DDC (0.5 µM) was incubated with 2 mM L-Dopa and 10 µM PLP in a final volume of 250 µL. The reaction was stopped by heating to 100 °C for 1 min. To enable extraction of the formed dopamine, 1 mL of 2,4,6-trinitrobenzenesulfonic acid (TNBSA) at 4.3 mM and 1.5 mL of benzene was added to the reaction mixture to enable conjugation of the formed dopamine with TNBSA. The trinitrophenylamine derivative was extracted into the benzene layer with 1 h of continuous shaking at 42 °C (275 RPM). The benzene layer was then collected, and the product formation was measured spectrophotometrically at 340 nm.

### 3.6. Biophysical Binding Studies

#### 3.6.1. Isothermal Calorimetry (ITC)

All ITC studies were conducted using a MICROCAL PEAQ-ITC automated system at the Institute for Structural Biology, Drug Discovery and Development (ISB3D) at VCU. ITC provides quantitative information on the binding affinity (*K*_d_), enthalpy changes (ΔH), and stoichiometry (n) of the interaction between two or more molecules in solution. The Gibbs free energy changes (ΔG) and entropy changes (ΔS) may be estimated from these initial observations using the following equation:

ΔG = RTln*K*_d_ = ΔH − TΔS (1)

Both the cell and titrant samples were co-dialyzed in 50 mM potassium phosphate, pH 7.4, containing 150 mM NaCl. For PNPO titration to apoDDC, apoDDC was prepared as described earlier and was dialyzed in 50 mM potassium phosphate solution, pH 7.4, containing 150 mM NaCl and 5% glycerol for 12 h, followed by a second dialysis for 4 h in the same buffer without glycerol. Table 1 illustrates the ITC parameters set for this experiment.

#### 3.6.2. Surface Plasmon Resonance (SPR)

Biacore T200, at the Biacore Molecular Interaction Shared Resource BMISR (Georgetown University, USA), was utilized to conduct binding experiments of PNPO and DDC. Three recombinant enzymes, apoDDC, holoDDC, and holoSHMT (rabbit cytoplasmic), were employed as ligands to immobilize on the CM5 chip surface using standard amine coupling chemistry. Samples were placed in flow cells (FCs) 2 to 4, with FC1 serving as a reference for FC2-4, and analyses were carried at 25 °C. The protein to be immobilized was diluted (1:50 dilution, 0.02 mg/mL diluted concentration) in 10 mM sodium acetate buffer at pH 5.5 and immobilized onto flow cell 2 (FC2) to a level of ~12,800 RU, flow cell 3 (FC3) to a level of ~10,100 RU, and flow cell 4 (FC4) to a level of ~9800 RU for apoDDC, holoDDC, and holoSHMT, respectively. PBS-P (10 mM phosphate buffer pH 7.4, 140 mM NaCl, 3 mM KCl, and 0.05% *v*/*v* surfactant P20) was used as the immobilization running buffer. Overnight kinetics were performed for analytes (PNPO and albumin) in the presence of PBS-P. One 15 s pulse of 1 M NaCl was injected for the surface regeneration and the flow rate of all analytes was maintained at 50 μL/min. The contact and dissociation times used for all analytes were 60 s and 300 s, respectively. The concentrations of the analytes used ranged from 100 μM to 1.5625 μM (2-fold dilutions), and each analyte was injected in triplicate.

### 3.7. PNPO•PLP Complex Preparation

The hPNPO enzyme and PLP (Sigma-Aldrich, St. Louis, MO, USA) complex was prepared as described previously by mixing at 1:5 molar ratio and incubation for 30 min at room temperature [8]. The mixture was then loaded onto a Sephadex G50 (0.6 × 45 cm) gel filtration column that had been pre-equilibrated with a 100 mM potassium phosphate buffer (pH 7.4) solution containing 5 mM 2-mercaptoethanol. The equilibration buffer was then passed through the column to elute any unbound PLP from the complex. The complex was further dialyzed overnight at 4 °C in the same buffer. The concentration of PNPO was determined by measuring the UV absorbance at 280 nm. The formed hPNPO-PLP complex was previously shown to have a stoichiometry of 1:1 (1 PLP bound per subunit) [6,8,10].

### 3.8. PLP Transfer from PNPO•PLP to Activate apoDDC

PLP transfer to activate apoDDC was monitored by a spectrophotometric assay described above. In this assay, a free PLP and PNPO•PLP complex (used as a PLP source) were utilized to convert the inactive apoDDC to the active holoDDC form. First, in 100 mM potassium phosphate (pH 7.4) at 25 °C, an equimolar amount of free PLP (50 µM) or PNPO•PLP (50 µM) was mixed with apoDDC (50 µM) at various times (2.5, 5, 10, 30, 60, and 90 min). Subsequently, the mixture was diluted (100-fold dilution) into the same buffer containing 2 mM of L-Dopa (substrate) to a final volume of 1 mL. Then, 250 µL aliquots were collected at different time intervals (0, 2, 6, and 10 min) and resuspended in 0.1 M perchloric acid solution (10% *v*/*v*) to stop the reaction. The product formed, dopamine, was determined by extracting and measuring the absorbance at 340 nm, as previously described. All experiments were run in at least triplicate.

## 4. Conclusions

This study was initiated to gain insight into how apo-B6 enzymes are converted to the catalytically active holo-forms. The traditional paradigm for PLP transfer to apo-B6 enzymes is for PLP to be released from PLK or PNPO into the bulk solvent and then acquired by the B6 enzymes. However, this proposal lacks an explanation for why the free PLP level is low in vivo, which might not meet the demand of newly synthesized apo-B6 enzymes for the cofactor. Additionally, releasing free PLP into the bulk solvent allows it to be degraded by PLP phosphatase to prevent toxic build-up of labile free PLP. Therefore, a second possibility is that the salvage and the B6 enzymes form a complex to sequester the PLP to prevent it from coming into contact with other cellular proteins or molecules, and allow productive transfer of PLP from the salvage enzymes to apo-B6 enzymes. Studies by our group and others have supported the second hypothesis [6,10,11,12,13,14]. However, attempts to crystallographically characterize the mode of interaction between the two enzymes have failed, presumably due to the transient nature of the interaction. Availability of the 3D protein structures of PNPO and DDC (in both the active state or holo-form and inactive state or apo-form) enabled us to undertake protein–protein docking and molecular dynamics simulation studies to predict the most likely near-native structure of the complex. Remarkably and consistently with our longstanding prediction, the interface of the putative complex is formed by the allosteric PLP tight binding site on hPNPO and the active site of DDC, providing evidence to support the role of the tight-binding PLP in the activation of apo-B6 enzymes. Moreover, the fact that PNPO recognized just one site of the asymmetric dimeric structure of apoDDC is also consistent with partial activation of apo-B6 enzymes by PNPO●PLP [8,12]. It is also clear that further research into site-directed mutagenesis is warranted to corroborate the putative complex. From the foregoing, the importance of vitamin B6 in several cellular processes and in the onset of pathologies as a result of PLP deficiency should also be looked at from the perspective of whether the delivery mechanism involving physical interactions between the salvage and B6 enzymes for productive transfer of free PLP is working appropriately. Clearly, in vivo inborn errors in the salvage enzymes that disrupt such interactions could prevent B6 activation with dire consequences. The putative complex is a step forward for understanding the pathological roles of identified mutations associated with pathological disorders and, perhaps, offers better therapeutic interventions.

## Figures and Tables

**Table 1:** ITC parameters set for PNPO•DDC titration.

ITC Parameters	Description
Total no. of injections	19
Cell temperature °C	25
Reference power (µcal/s)	5
Stir speed	750
Volume (µL)	2
Duration (s)	4
Spacing (s)	150

## Data Availability

Data are available on request.

## Supplementary Materials

The supporting information can be downloaded at: https://www.mdpi.com/article/10.3390/ijms24010642/s1.
