# Supplementary material for: Elucidating the Interaction between Pyridoxine 5′-Phosphate Oxidase and Dopa Decarboxylase: Activation of B6-Dependent Enzyme
Source: Int J Mol Sci. 2022 Dec 30;24(1):642. doi: 10.3390/ijms24010642 (PMC9820991; doi:10.3390/ijms24010642)
Supplement: Supplementary file 1 [file ijms-24-00642-s001.zip › ijms-2111648-supplementary.pdf]

SUPPORTING INFORMATION

Elucidating the Interaction between Pyridoxine 5′-Phosphate Oxidase and Dopa Decarboxylase: Activation of B6-Dependent Enzyme

Mohammed H. AL Mughram, Mohini S. Ghatge, Glen E. Kellogg and Martin K. Safo *

Department of Medicinal Chemistry and the Institute for Structural Biology, Drug Discovery, and Development, School of Pharmacy, Virginia Commonwealth University, Richmond, VA 23298, USA
* Correspondence: msafo@vcu.edu

This Document:

Table S1. Cluspro2.0 docking results for the top 10 clusters of PNPO and apoDDC (PDB 3RCH) complex, along with calculated HINT scores.

Table S2. Cluspro2.0 docking results for the top 10 clusters of PNPO and apoDDC-AF (model) complex, along with calculated HINT scores.

Table S3. Cluspro2.0 docking results for the top 10 clusters of PNPO and holoDDC complex (PDB 1JS3), along with calculated HINT scores.

Table S4. Cluspro2.0 docking results for the top 10 clusters of PNPO and holoDDC (model) complex, along with calculated HINT scores.

Table S5. Key interactions at the domain interface for the PNPO•apoDDC complex model as predicted by MD simulations.

Table S6. Key interactions at the domain interface for the PNPO•holoDDC complex model as predicted by MD simulations.

Table S7. *In silico* alanine scanning of the PNPO•apoDDC-AF complex obtained by hierarchical clustering of MD trajectories.

Figure S1. SPR results for the binding of PNPO to immobilized holoDDC and holoSHMT.

Table S1. Cluspro2.0 docking results for the top 10 clusters of PNPO and apoDDC (PDB: 3RCH) complex, along with calculated HINT scores.

Cluster	Members	%	HINT score	Hydrogen Bond	Acid/Base	Hydrophobic	Acid/Acid	Base/Base	Hydroph./Polar
0	76	7.6	2032.209	8596.166	3226.822	2706.76	-789.2139	-3385.264	-8323.062
1	71	7.1	1152.42	9578.741	2970.288	2839.457	-645.8171	-4312.954	-9277.295
2	43	4.3	6336.489	13193.75	3990.281	2181.616	-904.4463	-4329.23	-7795.483
3	37	3.7	2868.208	6721.397	1398.719	2037.818	-134.4676	-1730.892	-5424.368
4	36	3.6	7867.385	17079.55	4806.177	2496.075	-1252.961	-5930.431	-9331.025
5	35	3.5	7337.191	15509.96	3977.718	3029.297	-1070.994	-4849.986	-9258.809
6	32	3.2	3616.5	10424.32	2387.766	1955.174	-723.9514	-3269.073	-7157.736
7	32	3.2	4732.194	13330.25	4406.887	2810.313	-815.2784	-6449.8	-8550.173
8	29	2.9	5496.712	16148.84	4427.738	3060.189	-1024.375	-5939.218	-11176.46
9	26	2.6	-339.0726	7988.533	2497.652	2166.552	-623.7452	-4173.083	-8194.98

Table S2. Cluspro2.0 docking results for the top 10 clusters of PNPO and apoDDC-AF (model) complex, along with calculated HINT scores.

Cluster	Members	%	HINT score	Hydrogen Bond	Acid/Base	Hydrophobic	Acid/Acid	Base/Base	Hydroph./Polar
0	52	5.2	8649.985	13017.68	1870.801	1606.691	-733.7706	-2387.735	-4723.683
1	45	4.5	1789.329	10401.7	3242.745	2781.445	-505.138	-4667.735	-9463.684
2	44	4.4	549.0074	4843.478	2555.973	2012.546	-557.3123	-2878.877	-5426.8
3	43	4.3	3389.987	9625.224	2650.935	2134.361	-565.8962	-4081.872	-6372.764
4	40	4	745.7246	5119.332	1937.121	2381.634	-291.5302	-3445.69	-4955.142
5	36	3.6	2669.604	9728.706	2697.509	2230.926	-206.4271	-5673.592	-6107.517
6	34	3.4	668.2127	3655.788	1401.505	1523.839	-217.4465	-1175.301	-4520.171
7	30	3	5486.292	13080.61	3581.48	2238.726	-556.8046	-4326.55	-8531.171
8	29	2.9	2146.309	6029.135	1373.44	1534.223	-240.0219	-1718.776	-4831.691
9	29	2.9	348.5145	8042.262	2605.485	2045.279	-559.587	-4772.616	-7012.308

Table S3. Cluspro2.0 docking results for the top 10 clusters of PNPO and holoDDC complex (PDB 1JS3), along with calculated HINT scores.

Cluster	Members	%	HINT score	Hydrogen Bond	Acid/Base	Hydrophobic	Acid/Acid	Base/Base	Hydroph./Polar
0	58	5.8	2222.017	5653.545	1865.784	1384.034	-698.7088	-1508.832	-4473.805
1	55	5.5	2401.835	5388.355	2648.495	1539.826	-533.5378	-2249.115	-4392.188
2	52	5.2	2085.665	5640.911	2166.836	1396.976	-759.2861	-2337.534	-4022.238
3	47	4.7	11061.66	21090.79	4964.848	1864.776	-1026.855	-6830.125	-9001.772
4	45	4.5	859.9533	4174.195	2288.513	1464.489	-802.5218	-2194.99	-4069.731
5	44	4.4	11544.62	19866.46	4463.452	1803.829	-778.8778	-5986.074	-7824.172
6	44	4.4	13924.61	24358.32	5873.867	1794.173	-1151.033	-7894.123	-9056.592
7	33	3.3	9606.528	18262.32	5231.149	1945.587	-838.3188	-6069.647	-8924.565
8	29	2.9	-2173.584	5026.435	2564.959	1957.44	-817.2881	-3772.891	-7132.24
9	26	2.6	3512.605	11744.63	3275.028	1964.022	-1030.814	-4382.392	-8057.873

Table S4. Cluspro2.0 docking results for the top 10 clusters of PNPO and holoDDC (model) complex, along with calculated HINT scores.

Cluster	Members	%	HINT score	Hydrogen Bond	Acid/Base	Hydrophobic	Acid/Acid	Base/Base	Hydroph./Polar
0	78	7.8	4745.655	11350.68	3113.448	2308.501	-660.892	-4317.154	-7048.932
1	74	7.4	4755.787	11107.45	3106.383	2093.718	-882.9797	-3577.388	-7091.4
2	32	3.2	181.1883	4154.712	1557.112	1461.816	-168.1234	-2036.553	-4787.775
3	30	3	8928.14	14843.65	3738.476	1728.813	-807.2473	-3650.755	-6924.799
4	30	3	7503.392	14103.93	3613.991	1741.297	-585.3145	-4067.964	-7302.544
5	28	2.8	5248.711	10989.66	2477.844	1664.984	-412.624	-4168.736	-5302.421
6	26	2.6	3973.426	9907.376	2247.175	1939.506	-331.0345	-3082.377	-6707.22
7	23	2.3	4787.83	10147.88	2238.768	1874.134	-267.4978	-3531.957	-5673.502
8	19	1.9	3520.338	8087.063	2091.333	1954.558	-432.3741	-3062.331	-5117.911
9	19	1.9	2944.349	7515.712	2115.851	1722.862	-265.2529	-2371.483	-5773.34

Table S5. Key interactions at the domain interface for the PNPO•apoDDC complex model as predicted by MD simulations.

Donor	Acceptor	Occupancy
ARG88-sc	GLU421-sc	69.30%
ARG88-sc	ASP442-sc	67.93%
GLU114-sc	LYS207-sc	53.60%
TYR183-sc	GLU181-sc	47.62%
HIS439-sc	ARG88-sc	41.23%
TYR332-sc	GLN214-sc	36.13%
ARG197-sc	THR238-bb	36.10%
GLU181-sc	LYS187-sc	27.66%
ARG228-sc	GLU114-sc	20.30%
GLU480-sc	LYS119-sc	14.61%
GLU227-sc	LYS204-sc	13.70%

DDC chainA
DDC chainB
PNPO chainA
PNPO chainB

Table S6. Key interactions at the domain interface for the PNPO•holoDDC complex model as predicted by MD simulations.

Donor	Acceptor	Occupancy
THR246-sc	ASP240-sc	66.14%
SER193-sc	ASP240-sc	59.64%
THR238-sc	SER104-sc	58.89%
LYS91-sc	GLU425-sc	43.82%
SER193-bb	ASP240-sc	35.91%
THR54-sc	TYR332-bb	35.48%
ARG258-sc	ASP228-sc	35.35%
ARG426-sc	ASP89-sc	32.66%
TYR332-bb	LEU53-bb	28.24%
LYS327-sc	GLU251-sc	26.41%
ARG439-sc	GLU257-sc	17.26%
ARG347-sc	ASP253-sc	13.01%

DDC chainA
DDC chainB
PNPO chainA
PNPO chainB

Table S7. *In silico* alanine scanning of the PNPO•apoDDC-AF complex model obtained by hierarchical clustering of MD trajectories. Binding energy changes ($\Delta\Delta G = \Delta G_{wild\text{-}type} - \Delta G_{ALA}$) for key interacting amino acid residues at the interface with respect to clusters as predicted by Robetta server, where negative $\Delta\Delta G$ values (kcal mol^{-1}) indicate unfavorable replacement.

Residue no.	chainID	ΔΔG (kcal/mol)								AV	STDDEV
		cluster1	cluster2	cluster3	cluster4	cluster5	cluster6	cluster7	cluster8		
**103**	**DDC_B**	0.38		0.63	0.67	0.63				0.5775	0.13301
**176**	**DDC_B**	0.96	1.1	0.4		1.14	0.22			0.764	0.42459
**326**	**DDC_B**		0.34				0.23	0.24		0.27	0.06083
**327**	**DDC_B**	0.29	1.6	1.47	0.28	0.22	0.3	0.38	0.66	0.65	0.56338
**328**	**DDC_B**	0.66	0.99	0.83		0.62	0.65	0.82	0.6	0.7386	0.14462
**331**	**DDC_B**	0.4	0.49	0.31	0.21	0.46	0.3	0.13	0.36	0.3325	0.12186
**332**	**DDC_B**	2.42	2.29	2.68	2.47	2.72	2.47	2.42	2.56	2.5038	0.14292
**333**	**DDC_B**	0.36	0.27	0.33			0.27			0.3075	0.045
**353**	**DDC_B**	0.61		0.69	1.27	1.2	1.06	0.92	0.83	0.94	0.24993
**181**	**DDC_A**		0.7	1.27	0.97	0.17	-0.07			0.608	0.55436
**197**	**DDC_A**	3.69	2.95	2.32	2.66	3.37	2.8	2.29	2.95	2.8788	0.48144
**200**	**DDC_A**	0.95	0.97	0.66	0.68	2.4	0.83	1.04	0.64	1.0213	0.57791
**201**	**DDC_A**	0.34	0.52					0.54		0.4667	0.11015
**207**	**DDC_A**	0.51	1.51	1.83	2.4		0.98	2.65	0.77	1.5214	0.81871
**228**	**DDC_A**	1.14	0.33		0.96	1.08	1.18	2.11	0.31	1.0157	0.60764
**417**	**DDC_A**	1.03	0.61	0.93	0.98	0.65	3	1.92	0.54	1.2075	0.84468
**421**	**DDC_A**	0.77	4.15	0.59	1.47	1.05	1.45	2.7	3.14	1.915	1.27286
**441**	**DDC_A**		0.11	0.1	0.06	0.12	0.01	0.03	0.12	0.0786	0.04525
**442**	**DDC_A**	0.57	-0.48	0.66	0.6	0.99	1.3	0.58	0.41	0.5788	0.51352
**51**	**PNPO_B**	0.08		0.23		0.36				0.2233	0.14012
**56**	**PNPO_B**		1.06	1.11		0.43				0.8667	0.37899
**88**	**PNPO_B**	1.37	4.59	1.06	1.95	1.49	1.79	3.02	3.49	2.345	1.23077
**119**	**PNPO_B**	0.13			0.4		0.67	0.51	0.05	0.352	0.25927
**212**	**PNPO_B**	1.09	0.67	1.18		0.96	1.05	1.06	0.78	0.97	0.18221
**214**	**PNPO_B**	0.21	0.76	0.83	0.68	0.92	0.43	0.52	0.71	0.6325	0.23249
**234**	**PNPO_B**	0.33	0.39	0.38	0.41	0.51	0.34	0.39	0.47	0.4025	0.06112
**236**	**PNPO_B**	0.48	0.67	0.72	0.75	0.23	0.79	0.52	0.32	0.56	0.20743
**238**	**PNPO_B**	0.23			0.35	0.39				0.3233	0.08327
**240**	**PNPO_B**	0.99	0.52	-0.21	-0.14		0.24	-0.11	-0.18	0.1586	0.45466
**241**	**PNPO_B**		0.96	-0.01	-0.03	-0.08	-0.05	-0.05		0.1233	0.41054
**249**	**PNPO_B**		1.34	0.72	-0.11	0.15	0.91		0.21	0.5367	0.54639
**252**	**PNPO_B**	-0.07		-0.08	0.28			0.42		0.1375	0.25198
**253**	**PNPO_B**	0.2	-0.17		0.34	0.15	0.49	-0.09	0.82	0.2486	0.34036
**88**	**PNPO_A**	1.31	0.33	1.34	1.18	1.74	2.12	1.53	1.57	1.39	0.51879
**114**	**PNPO_A**	0.98	0.27	0.03	1.44	1.71	1.55	2.32	0.52	1.1025	0.7895
**119**	**PNPO_A**	0.54	0.08	0.24	0.69		0.73	-0.43	0.32	0.31	0.40398
**183**	**PNPO_A**		0.84	0.64	1.18	-0.04	0.22			0.568	0.48592
**187**	**PNPO_A**		-0.15	0.45		0.2				0.1667	0.30139
**204**	**PNPO_A**	0.95	0.77	0	0	0.57	0.13	0.16	1.02	0.45	0.42815

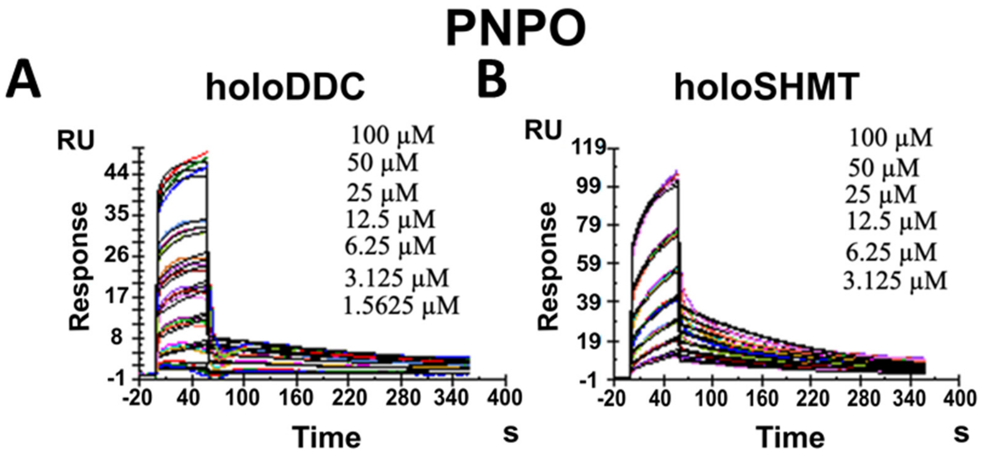

K_a (1/Ms)	K_d (1/s)	K_D (µM)	Chi^2	U-Value
868	0.00322	3.7	0.722	5

K_a (1/Ms)	K_d (1/s)	K_D (µM)	Chi^2	U-Value
366	0.00565	15.5	3.06	2

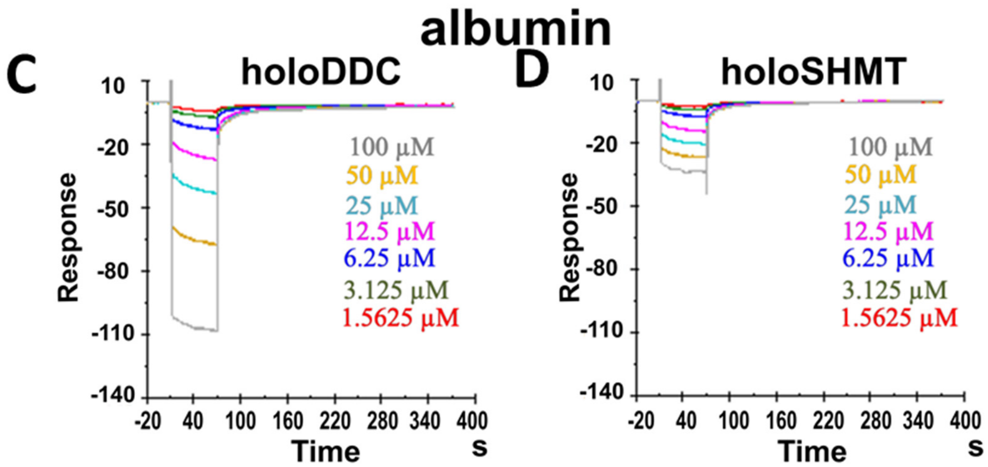

Figure S1. SPR results for the binding of PNPO to immobilized holoDDC and holoSHMT. (A) SPR sensorgram binding of PNPO to holoDDC; (B) SPR sensorgram binding of PNPO to holoSHMT (positive control); and (C-D) Negative control, binding of albumin to holoDDC and holoSHMT, respectively. Flow rate of all analytes were maintained at 50 µL/min, and injections of 100 µM to 1.5625 µM (from top to bottom) were made. The contact and dissociation times used for all analytes were 60 s and 300 s, respectively. One 15 s pulse of 1 M NaCl was injected for surface regeneration. All analytes were injected in triplicate.
